# Central Nervous System Activity of Lazertinib in *EGFR* ‐Mutated NSCLC With Prior EGFR TKI Exposure: A Pooled Analysis of KCSG‐LU20‐15 and LU21‐01

**DOI:** 10.1111/1759-7714.70387

**Published:** 2026-08-31

**Authors:** Dong Hyun Kim, Min Hee Hong, Hyun Ae Jung, Tae Min Kim, Chang Gon Kim, Hee Kyung Ahn, Youngjoo Lee, Yu Jung Kim, Miso Kim, Jeonghwan Youk, Jong‐Mu Sun, Se‐Hoon Lee, Jin Seok Ahn, Dong‐Wan Kim, Myung‐Ju Ahn, Yoon Ji Choi, Sun Min Lim, Jin Hyoung Kang, Bhumsuk Keam, Hye Ryun Kim

**Affiliations:** ^1^ Department of Internal Medicine Seoul National University Hospital Seoul Republic of Korea; ^2^ Department of Translational Medicine Seoul National University College of Medicine Seoul Republic of Korea; ^3^ Division of Medical Oncology, Department of Internal Medicine, Yonsei Cancer Center Severance Hospital, Yonsei University College of Medicine Seoul Republic of Korea; ^4^ Division of Hematology and Oncology, Department of Medicine Samsung Medical Center, Sungkyunkwan University School of Medicine Seoul Republic of Korea; ^5^ Cancer Research Institute Seoul National University College of Medicine Seoul Republic of Korea; ^6^ Division of Medical Oncology Gachon University Gil Medical Center Incheon Republic of Korea; ^7^ Center for Lung Cancer National Cancer Center Goyang Gyeonggi Republic of Korea; ^8^ Department of Internal Medicine, Seoul National University Bundang Hospital Seoul National University College of Medicine Seongnam Republic of Korea; ^9^ Division of Medical Oncology and Hematology, Department of Internal Medicine Korea University Anam Hospital, Korea University College of Medicine Seoul Republic of Korea; ^10^ Division of Medical Oncology, Department of Internal Medicine, Seoul St Mary's Hospital, College of Medicine The Catholic University of Korea Seoul Republic of Korea

**Keywords:** CNS, EGFR, lazertinib, leptomeningeal metastasis, NSCLC, pooled analysis

## Abstract

**Background:**

Central nervous system (CNS) involvement remains a major cause of mortality in patients with epidermal growth factor receptor (EGFR)‐mutated nonsmall cell lung cancer (NSCLC), particularly, after progression on EGFR tyrosine kinase inhibitors (TKIs). This pooled analysis evaluated the efficacy of lazertinib in patients with *EGFR*‐mutated NSCLC and CNS involvement, using data from two Phase II trials.

**Patients and Methods:**

Patients with *EGFR*‐mutated NSCLC and CNS involvement following prior EGFR TKI therapy enrolled in the KCSG‐LU20‐15 and KCSG‐LU21‐01 were included. Overall and intracranial objective response rate (ORR), disease control rate (DCR), and progression‐free survival (PFS) were assessed.

**Results:**

Seventy‐two patients were included. The median age was 63 years and 45 patients (62.5%) had leptomeningeal metastases (LM). T790M status was positive in 7 patients, negative in 44, and unknown in 21. Intracranial tumor responses were evaluable in 46 patients; the intracranial ORR was 50.0%, and the intracranial DCR was 97.8%. The median intracranial PFS was 15.2 months. Among 66 patients evaluable for systemic response, the overall ORR and DCR were 22.7% and 97.0%, respectively. The median PFS and overall survival were 10.6 and 17.8 months, respectively. Patients with LM experienced inferior OS and intracranial PFS compared with those without LM. No significant differences in clinical outcomes were observed according to T790M status.

**Conclusion:**

Lazertinib demonstrated meaningful CNS activity in patients with *EGFR*‐mutated NSCLC and CNS involvement after prior EGFR TKI exposure, including those with LM. Notably, this activity was observed in a cohort with a low prevalence of confirmed T790M positivity.

## Graphical abstract

This pooled analysis of two Phase II trials included 72 patients with EGFR‐mutated NSCLC and CNS involvement after prior EGFR TKI exposure. Lazertinib achieved an intracranial ORR of 50.0%, with median intracranial PFS and OS of 15.2 and 17.8 months, respectively, supporting meaningful CNS activity in this later‐line setting.

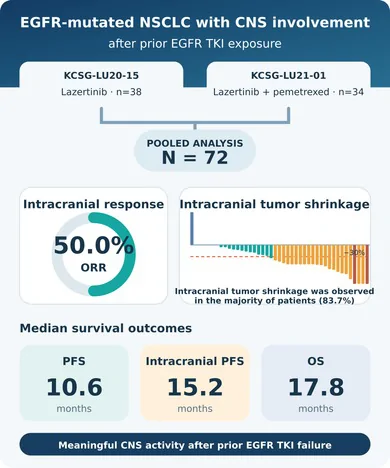

## 1 Introduction

Epidermal growth factor receptor (EGFR)‐mutated nonsmall cell lung cancer (NSCLC) represents a distinct molecular subtype characterized by high initial sensitivity to tyrosine kinase inhibitors (TKIs) [1], yet remains clinically challenged by resistance and an elevated risk of central nervous system (CNS) failure [2]. CNS involvement, including brain metastases and leptomeningeal metastases (LM), constitutes a major cause of morbidity and mortality, largely owing to incomplete CNS penetration of first‐ or second‐generation EGFR TKIs and limited efficacy after CNS progression [3].

Lazertinib is a potent, third‐generation EGFR TKI with favorable blood–brain barrier permeability [4]. Across early‐phase and randomized studies, lazertinib has demonstrated clinically meaningful efficacy against both systemic and intracranial disease, supporting its role as a CNS‐active EGFR TKI [5]. Recent data have further suggested promising CNS activity of lazertinib in treatment‐naïve patients with *EGFR*‐mutated NSCLC [6]. However, outcomes remain suboptimal in patients with CNS progression following prior exposure to EGFR TKIs. Pooled analyses incorporating treatment‐naïve populations alongside previously treated patients may not fully reflect the clinical scenario of CNS progression after prior EGFR TKI therapy. A focused evaluation in patients with prior EGFR TKI treatment may therefore offer complementary insight into the therapeutic role of lazertinib in this setting.

We conducted two Phase II trials of later‐line lazertinib in *EGFR*‐mutated NSCLC with CNS involvement [7, 8]. On this basis, we conducted a pooled analysis of two prospective Phase II trials to evaluate the efficacy of lazertinib in patients with *EGFR*‐mutant NSCLC with CNS involvement following prior EGFR TKI exposure, with a particular focus on intracranial outcomes. Pooling these cohorts increased the number of evaluable patients and enabled a broader assessment of CNS efficacy in this clinically challenging setting.

## 2 Patients and Methods

### 2.1 Study Design

**FIGURE 1:**
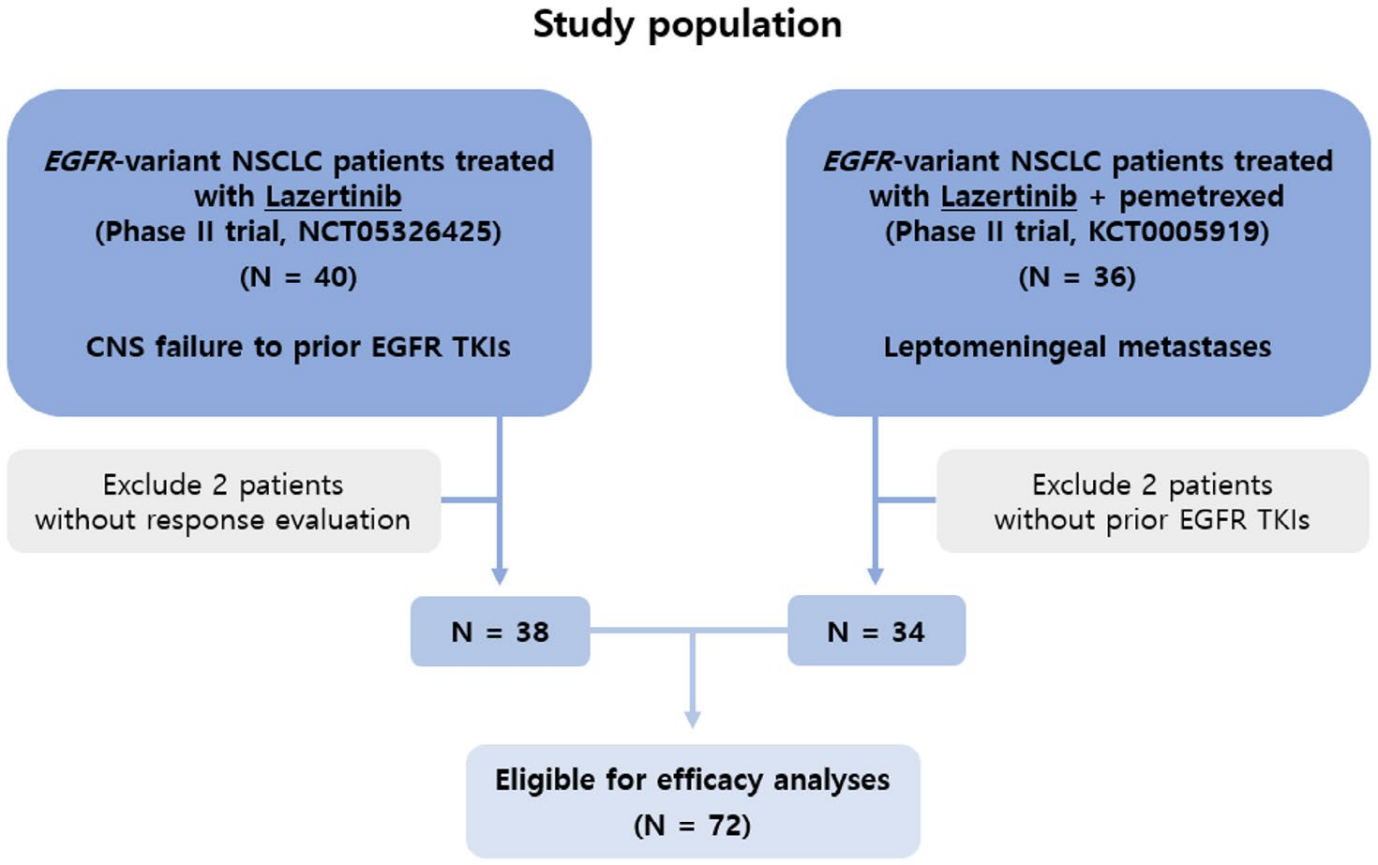
Consort diagram.

Data for this study were obtained from two prospective clinical trials that evaluated the clinical outcomes of lazertinib in patients with *EGFR*‐variant NSCLC and CNS metastases, with a focus on those with prior EGFR TKI exposure. Detailed descriptions of the study designs for these trials have been published previously [7, 8]. Briefly, the KCSG‐LU20‐15 trial (ClinicalTrials.gov identifier: NCT05326425) was a multicenter, single‐arm, Phase II study in which 40 patients were enrolled and treated with lazertinib [7]. After excluding two patients who were not evaluable for outcomes because of early termination, 38 patients were included in the analysis. The KCSG‐LU21‐01 trial (Clinical Research Information Service identifier: KCT0005919) was a multicenter, single‐arm, Phase II study in which 36 patients were enrolled [8]. Patients were treated with lazertinib in combination with pemetrexed. After excluding two patients who had not received prior EGFR TKI therapy, 34 patients were included in the analysis. Overall, the final analysis comprised 72 patients with prior EGFR TKI exposure who underwent response evaluation (Figure 1). The pooled analysis was intended to characterize CNS outcomes across the combined post‐EGFR TKI population rather than to compare the two treatment regimens. Accordingly, efficacy and survival outcomes were also analyzed separately by source trial. This study was approved by the Institutional Review Board of each participating center and was performed following the protocol, good clinical practice guidelines, and the Declaration of Helsinki. Informed consent was obtained from all patients before enrollment.

### 2.2 Patients and Treatments

In both the KCSG‐LU20‐15 and KCSG‐LU21‐01 cohorts, patients with histologically confirmed NSCLC and confirmed *EGFR* mutations were eligible. In KCSG‐LU20‐15, key inclusion criteria included brain metastases after failure of first‐ or second‐generation EGFR TKIs and the presence of measurable intracranial metastases. LM was permitted. In KCSG‐LU21‐01, cytologically confirmed LM was required, whereas patients without measurable lesions were permitted.

In both trials, lazertinib was administered at a dose of 240 mg once daily and was continued until objective disease progression, unacceptable toxicity, withdrawal of consent, or death. In both trials, treatment could be continued beyond disease progression at the investigator's discretion. The detailed dosing schedules for each clinical trial are provided in the Supporting Information Methods.

### 2.3 Assessments

The primary endpoints were intracranial objective response rate (iORR) in KCSG‐LU20‐15 and overall survival (OS) in KCSG‐LU21‐01. Tumor response was assessed by investigators using the Response Evaluation Criteria in Solid Tumors (RECIST) version 1.1. In KCSG‐LU20‐15, response assessments were performed every 6 weeks for the first four evaluations, followed by every 12 weeks thereafter. In KCSG‐LU21‐01, response was assessed every 9 weeks. The detailed response evaluation for each trial is described in the Supporting Information Methods.

In this pooled analysis, overall objective response rate (ORR) and disease control rate (DCR), progression‐free survival (PFS), and OS were assessed. CNS‐related clinical efficacy was also assessed, including iORR, intracranial DCR (iDCR), and intracranial PFS (iPFS). Intracranial response rates were evaluated in patients with baseline measurable CNS lesions. iPFS was evaluated in all patients and defined as the time from treatment initiation to the first occurrence of intracranial disease progression or death from any cause, whichever occurred first.

### 2.4 Statistical Analysis

Descriptive statistics are presented as median values with ranges or numbers with percentages. Differences between the groups were analyzed using Student's *t*‐test or Wilcoxon rank‐sum test for continuous variables and Fisher's exact test or Pearson's *χ* ^2^ test for categorical variables, as appropriate. The Clopper–Pearson method was used for calculating the 95% confidence interval (CI) for the response rate. Survival outcomes were estimated using the Kaplan–Meier method, and the log‐rank test was used to assess differences in survival outcomes between groups. The statistical software “R” version 4.4.1 (www.r‐project.org) was used for all statistical analyses. *p*‐values of < 0.05 were considered statistically significant.

## 3 Results

### 3.1 Patient and Disease Characteristics

In total, 72 patients with prior EGFR TKI exposure were included in the analysis, and their baseline characteristics are presented in Table 1. Among them, 38 were from the KCSG‐LU20‐15 trial, whereas the remaining 34 were from the KCSG‐LU21‐01 trial (Table S1). The median age was 63 years (range, 29–85), and 43 patients (59.7%) were female. At diagnosis, 40 patients (55.6%) harbored an *EGFR* exon 21 L858R mutation, and 29 patients (40.3%) had an exon 19 deletion. All patients had previously received first‐ or second‐generation EGFR TKI therapy, and five patients had been treated with two or more EGFR TKIs. Prior EGFR TKI treatments included gefitinib (47.2%), afatinib (43.1%), erlotinib (16.7%), and dacomitinib (1.4%). Overall, T790M mutation status was assessed in 51 patients, of whom 7 were T790M‐positive. Forty‐five patients (62.5%) had LM.

**TABLE 1:** Baseline characteristics.

Variables	*N* = 72
Age, years, median (range)	63 (29–85)
Age ≥ 65, *n* (%)	31 (43.1)
Sex, *n* (%)	
Male	29 (40.3)
Female	43 (59.7)
ECOG performance status, *n* (%)	
0	8 (11.1)
1	51 (70.8)
2	13 (18.1)
Smoking history, *n* (%)	
Never smoker	49 (68.1)
Ex‐smoker	22 (30.6)
Current smoker	1 (1.4)
Histology, *n* (%)	
Adenocarcinoma	67 (93.1)
Squamous cell carcinoma	2 (2.8)
Adenosquamous cell carcinoma	1 (1.4)
Nonsmall cell lung cancer, not otherwise specified	2 (2.8)
EGFR variants prior to previous EGFR TKIs, *n* (%)	
Exon 19 deletion	29 (40.3)
L858R	40 (55.6)
Others^a^	3 (4.2)
Prior EGFR TKI^b^, *n* (%)	
Gefitinib	34 (47.2)
Afatinib	31 (43.1)
Erlotinib	12 (16.7)
Dacomitinib	1 (1.4)
Prior lines of systemic treatment, *n* (%)	
1	43 (59.7)
2	20 (27.8)
≥ 3	9 (12.5)
T790M status, *n* (%)	
Positive	7 (9.7)
Negative	44 (61.1)
Unknown	21 (29.2)
Type of CNS lesion, *n* (%)	
Brain metastasis only	27 (37.5)
Leptomeningeal metastasis ± brain metastasis	45 (62.5)
Other metastatic sites, *n* (%)	
Bone	33 (45.8)
Lymph node	31 (43.1)
Pleura	11 (15.3)
Liver	5 (6.9)
Adrenal	3 (4.2)

Abbreviations: CNS, central nervous system; ECOG, Eastern Cooperative Oncology Group; EGFR, epidermal growth factor receptor; TKI, tyrosine kinase inhibitor.

^a^ Exon 18 (*n* = 1), L861Q (*n* = 1), and EGFR G719A/S768I (*n* = 1).

^b^ Five patients received more than one TKI, resulting in overlap.

### 3.2 Objective Response

**FIGURE 2:**
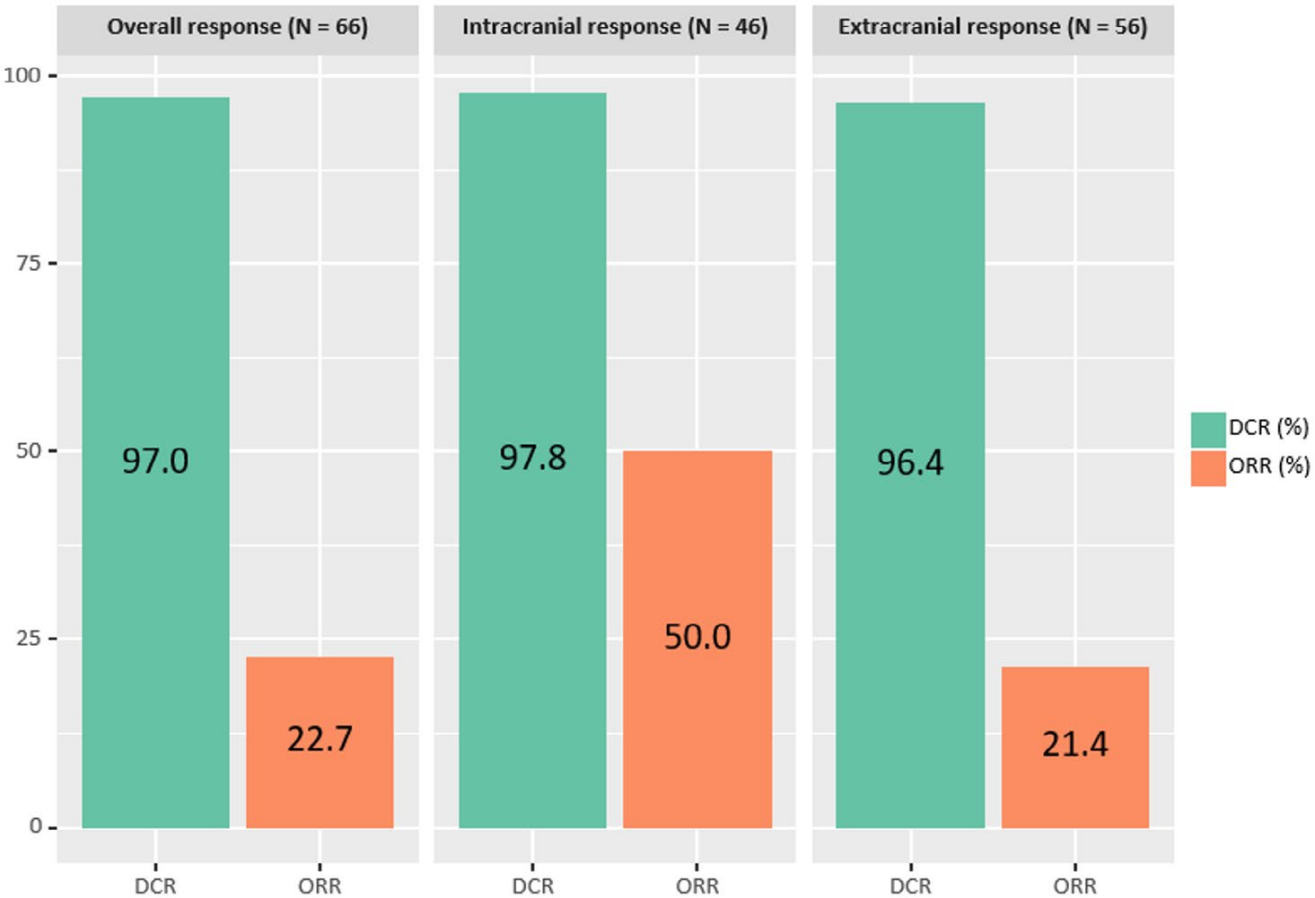
Response rates. DCR, disease control rate; ORR, objective response rate.

Overall, response rates were assessed in 66 patients with measurable lesions (Table 2). Based on the best response, 15 patients (22.7%) achieved a partial response (PR), 49 (74.2%) had stable disease (SD), and 2 (3.0%) had progressive disease (PD). The ORR was 22.7% (95% CI, 13.3–34.7), and the DCR was 97.0% (95% CI, 89.5–99.6) (Figure 2). Intracranial tumor responses were evaluable in 46 patients; 2 (4.3%) achieved a complete response (CR), 21 (45.7%) had PR, 22 (47.8%) had SD, and 1 (2.2%) had PD. The iORR was 50.0% (95% CI, 34.9–65.1), and the iDCR was 97.8% (95% CI, 88.5–99.9). No significant differences in ORR or iORR were observed between the individual study cohorts (Table S2). Extracranial tumor responses were evaluable in 56 patients; 12 (21.4%) had PR, 42 (75.0%) had SD, and 2 (3.6%) had PD. The extracranial ORR was 21.4% (95% CI, 11.6–34.4), and the extracranial DCR was 96.4% (95% CI, 87.7–99.6).

**TABLE 2:** Response assessments: overall, intracranial, and extracranial.

Variables	*N* = 66
Overall response, *n* (%)	
Complete response	0
Partial response	15 (22.7)
Stable disease	49 (74.2)
Progressive disease	2 (3.0)
Overall objective response rate, % (95% CI)	22.7 (13.3–34.7)
Overall disease control rate, % (95% CI)	97.0 (89.5–99.6)
Intracranial response, *n* (%)	*N* = 46
Complete response	2 (4.3)
Partial response	21 (45.7)
Stable disease	22 (47.8)
Progressive disease	1 (2.2)
Intracranial objective response rate, % (95% CI)	50.0 (34.9–65.1)
Intracranial disease control rate, % (95% CI)	97.8 (88.5–99.9)
Extracranial response, *n* (%)	*N* = 56
Complete response	0
Partial response	12 (21.4)
Stable disease	42 (75.0)
Progressive disease	2 (3.6)
Extracranial objective response rate, % (95% CI)	21.4 (11.6–34.4)
Extracranial disease control rate, % (95% CI)	96.4 (87.7–99.6)

Abbreviation: CI, confidence interval.

**FIGURE 3:**
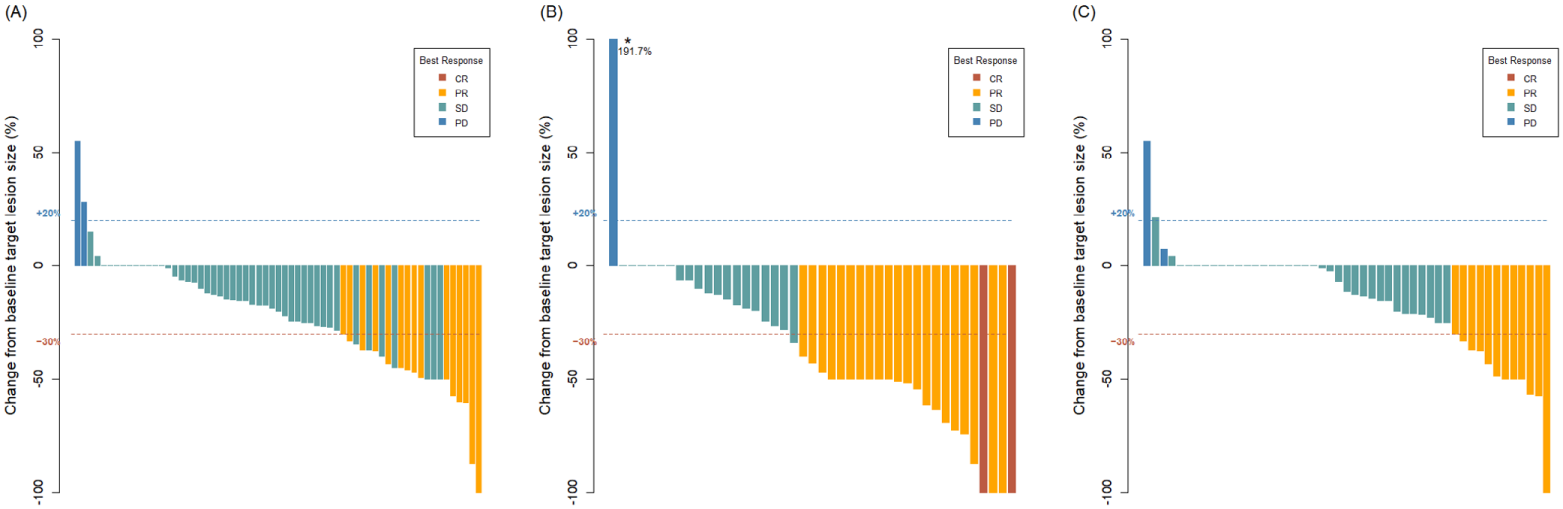
Waterfall plot of the best percentage change from baseline in target lesion size. (A) Overall response (*N* = 63); (B) intracranial response (*N* = 43); (C) extracranial response (*N* = 49).

A waterfall plot illustrates the percentage change from baseline in tumor burden of target lesions for each patient. Of 63 patients, 49 (77.8%) showed a reduction in overall tumor burden, and 9 patients experienced a reduction of 50% or greater (Figure 3A). Intracranial tumor shrinkage was observed in the majority of patients (83.7%), with best responses ranging from −100% to +191.7% (Figure 3B). A reduction of ≥ 50% was observed in nearly half of the patients, whereas intracranial tumor growth was observed in only one patient (2.3%). Regarding extracranial response, tumor shrinkage was observed in 57.1% of patients (Figure 3C).

### 3.3 Survival Outcomes

**FIGURE 4:**
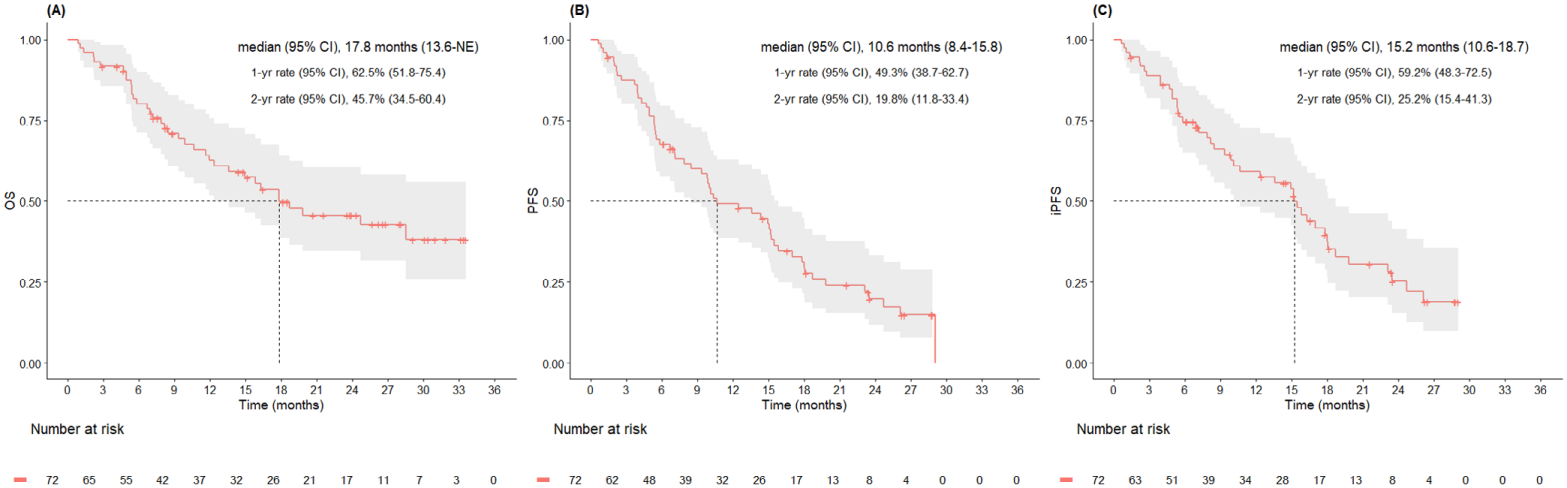
Kaplan–Meier curves of survival outcomes. (A) Overall survival; (B) progression‐free survival; (C) intracranial progression‐free survival.

The median follow‐up duration was 24.3 months (95% CI, 21.6–28.1) in the overall cohort; patients in the LU20‐15 had a median follow‐up of 26.2 months, whereas those in the LU21‐01 had a median follow‐up of 21.6 months. The median OS was 17.8 months (95% CI, 13.6–not estimable), with 1‐ and 2‐year OS rates of 62.5% (95% CI, 51.8–75.5) and 45.7% (95% CI, 34.5–60.5), respectively (Figure 4A). The median PFS was 10.6 months (95% CI, 8.4–15.8), with 1‐ and 2‐year PFS rates of 49.3% (95% CI, 38.7–62.7) and 19.8% (95% CI, 11.8–33.4), respectively (Figure 4B). The median iPFS was 15.2 months (95% CI, 10.6–18.7), with 1‐ and 2‐year iPFS of 59.2% (95% CI, 48.3–72.6) and 25.3% (95% CI, 15.4–41.3), respectively (Figure 4C). The LU21‐01 cohort, which consisted exclusively of patients with LM, showed poorer survival outcomes than the LU20‐15 cohort, in which 28.9% of patients had LM (Figure S1).

### 3.4 Exploratory Subgroup Analysis

No statistically significant differences were observed between the exon 19 deletion and L858R groups in OS (median, 28.6 vs. 17.8 months; hazard ratio [HR], 0.76; 95% CI, 0.38–1.53; *p* = 0.440), PFS (median, 10.6 vs. 12.4 months; HR, 0.80; 95% CI, 0.46–1.41; *p* = 0.450), or iPFS (median, 17.8 vs. 15.2 months; HR, 0.86; 95% CI, 0.46–1.59; *p* = 0.630) (Figure S2). According to CNS disease pattern, patients with LM exhibited significantly shorter OS (median, 11.9 months vs. not reached; HR, 6.10; 95% CI, 2.44–15.99; *p* < 0.001) and PFS (median, 9.9 vs. 17.8 months; HR, 1.93; 95% CI, 1.08–3.44; *p* = 0.024) (Figure S3). In addition, iPFS was significantly shorter in patients with LM (median, 10.1 vs. 23.1 months; HR, 2.35; 95% CI, 1.22–4.53; *p* = 0.009).

Among the 51 patients with known T790M status, clinical outcomes were compared between patients with T790M‐positive (*n* = 7) and T790M‐negative disease (*n* = 44). No significant differences were observed between the two groups in overall ORR, iORR, or extracranial ORR (Table S3). Similarly, there were no significant differences in OS (median, 18.7 months vs. not reached; HR, 0.49; 95% CI, 0.11–2.06; *p* = 0.320), PFS (median, 10.1 vs. 13.6 months; HR, 1.53; 95% CI, 0.67–3.51; *p* = 0.310), or iPFS (median, 14.9 vs. 15.8 months; HR, 1.40; 95% CI, 0.53–3.68; *p* = 0.490) (Figure S4).

## 4 Discussion

In this pooled analysis of the KCSG‐LU20‐15 and LU21‐01 studies, lazertinib demonstrated encouraging clinical activity in patients with CNS involvement and prior EGFR TKI exposure. Overall, the iORR was 50.0% and median iPFS was 15.2 months. In a cohort with a substantial proportion of patients with LM, lazertinib demonstrated meaningful intracranial responses and durable CNS disease control, supporting its role as a CNS‐active systemic therapy following prior EGFR TKI treatment.

Third‐generation EGFR TKIs are active against the T790M mutation, the most common resistance mechanism following failure of first‐ or second‐generation EGFR TKIs, and exhibit robust activity within the CNS [9]. In the Phase I/II LASER201 study, lazertinib demonstrated promising efficacy in patients with *EGFR*‐mutated NSCLC, with improved survival outcomes observed among those with T790M‐positive tumors [5]. In the dose extension cohort of the LASER201 study, first‐line lazertinib achieved an overall ORR of 70% and a median PFS of 24.6 months [10]. Among patients with baseline brain metastases, both median OS and iPFS were not reached at the time of analysis. Preclinical models showed that lazertinib penetrates the blood–brain barrier and suppresses intracranial tumor growth, supporting its CNS activity [11]. Subsequently, the randomized Phase III LASER301 study confirmed the efficacy of lazertinib in the first‐line setting, demonstrating prolonged PFS compared with gefitinib [12]. Importantly, CNS subgroup analyses from LASER301 showed consistent benefit irrespective of baseline brain metastasis status [13, 14, 15]. In a pooled analysis focusing on CNS activity from the LASER201 and LASER301 studies, lazertinib achieved an iORR of 91.7% and a median iPFS of 27.7 months in the first‐line setting [6]. In the prior EGFR TKI‐treated cohort of the LASER201 study, lazertinib achieved an iORR of 85.7% and a median iPFS of 26.0 months [16]. However, all patients were T790M‐positive, only seven had measurable intracranial lesions, and eligibility was limited to asymptomatic brain metastases, excluding patients with symptomatic disease or LM. Our study focused on a post‐EGFR TKI population with CNS involvement, including a high proportion of patients with LM (62.5%), thereby complementing existing evidence and suggesting that the CNS activity of lazertinib may be preserved beyond the first‐line setting.

In parallel, the CNS efficacy of osimertinib, another third‐generation EGFR TKI, has been extensively characterized across multiple trials. In pooled analyses of the AURA extension and AURA2 Phase II trials in patients with T790M‐positive NSCLC and stable brain metastases after prior EGFR TKI treatment, osimertinib achieved an iORR of 54% with durable iPFS [17]. In the Phase I BLOOM study, osimertinib achieved an iORR of 62% and a median PFS of 8.6 months in patients with LM following prior EGFR TKI therapy [18]. In the AURA LM analysis, osimertinib achieved an LM ORR of 55% and a median OS of 18.8 months in patients with T790M‐positive NSCLC and LM who had progressed after prior EGFR TKI therapy [19]. In a separate Phase II study of osimertinib 160 mg, the LM cohort achieved an iDCR of 92.5% and a median OS of 13.3 months [20]. The CNS efficacy observed with lazertinib in our analysis falls within the range reported in previous osimertinib studies. However, these findings should not be interpreted as evidence of comparable efficacy, as any cross‐trial comparison is limited by substantial differences in eligibility criteria, patient characteristics, response‐assessment methods, and treatment regimens.

In contemporary clinical practice, particularly, with the widespread adoption of third‐generation EGFR TKIs in the frontline setting, the incidence of acquired T790M mutations after prior EGFR TKI therapy has declined. Accordingly, CNS progression increasingly occurs in the absence of identifiable T790M‐mediated resistance. In this context, our pooled cohort—characterized by a low prevalence of T790M positivity (< 10%) and a high burden of CNS involvement, including LM—represents a clinically relevant population reflective of current practice. In an exploratory analysis, outcomes did not differ significantly according to T790M status, suggesting that CNS activity of third‐generation EGFR TKIs may not be strictly dependent on T790M status. This concept is supported by previous evidence showing that osimertinib is active against LM even in cohorts not enriched for T790M. In the Phase II BLOSSOM study, which enrolled patients who developed LM after prior first‐ or second‐generation EGFR TKI therapy, most patients were T790M‐negative; nevertheless, the leptomeningeal ORR was 51.6%, with a median iPFS of 11.2 months [21]. However, evidence regarding their efficacy in T790M‐negative brain metastases after prior EGFR TKI therapy remains limited and heterogeneous [22, 23]. Our findings align with and extend emerging evidence that the CNS activity of third‐generation EGFR TKIs, including lazertinib, may be preserved beyond strictly T790M‐selected populations, while highlighting the need for further investigation.

The safety profiles of the two prospective studies have been reported in detail previously; therefore, a pooled safety analysis was not performed in the present study, which was primarily designed to evaluate CNS efficacy. Overall, the safety profiles reported in the two studies were generally similar, with no unexpected safety findings. In the LU21‐01 study, in which lazertinib (240 mg once daily, with dose escalation to 320 mg at the investigator's discretion) was administered in combination with pemetrexed, grade ≥ 3 adverse events occurred in 13.9% of patients. In comparison, grade ≥ 3 adverse events were reported in 10.0% of patients treated with single‐agent lazertinib (240 mg once daily) in the LU20‐15 study.

This study had several limitations. First, this was a post hoc pooled analysis of two single‐arm Phase II trials, and the absence of a control group precludes direct comparative conclusions. In addition, the sample size remains relatively limited, particularly, for subgroup analyses. Due to the small number of T790M‐positive patients, the study was not adequately powered to compare efficacy according to T790M status or to establish T790M‐independent CNS activity. Second, heterogeneity between the two trials—including differences in CNS disease composition and the use of combination therapy in one cohort—may have influenced the outcomes. Because the proportion of patients with LM differed substantially between the two cohorts, LM status was confounded with study cohort and treatment regimen. Therefore, survival comparisons between patients with and without LM should be interpreted with caution. Third, as third‐generation EGFR TKIs have become the standard of care, the proportion of patients previously treated with first‐ or second‐generation EGFR TKIs is expected to decline over time; consequently, the applicability of our findings may be limited. Finally, patient‐level safety data were not available for the present pooled analysis, precluding a regimen‐stratified assessment of treatment‐related adverse events, dose modifications, and treatment discontinuations.

Despite these limitations, our study provides evidence supporting the activity of lazertinib in patients with CNS involvement after prior EGFR TKI exposure. In a cohort in which more than 60% of patients had LM, the observed iORR of 50% and median iPFS of 15.2 months further reinforce the role of lazertinib as a CNS‐active EGFR TKI. In addition, the majority of patients in our cohort were T790M‐negative or had unknown T790M status, suggesting that the CNS activity of lazertinib may not be restricted to T790M‐positive populations.

## 5 Conclusion

In conclusion, findings from this pooled analysis from KCSG‐LU20‐15 and LU21‐01 trials further confirmed the CNS activity of lazertinib in patients with *EGFR*‐mutated NSCLC who had progressed after first‐ or second‐generation EGFR TKIs. These results demonstrate consistent outcomes across KCSG‐LU20‐15 and KCSG‐LU21‐01 studies and further support the CNS efficacy of lazertinib in patients with LM following prior EGFR TKI therapy. CNS activity was observed in a cohort with a low prevalence of confirmed T790M positivity; this observation is hypothesis‐generating and requires validation in prospectively defined T790M‐negative populations. Further studies with a larger sample size and translational analyses are warranted to optimize the treatment of LM in *EGFR*‐mutated NSCLC.

## Author Contributions

**Dong Hyun Kim:** writing – review and editing, writing – original draft, visualization, formal analysis, investigation, data curation, conceptualization. **Min Hee Hong:** writing – review and editing, writing – original draft, formal analysis, investigation, resources, data curation. **Hyun Ae Jung:** writing – review and editing, investigation, resources, data curation. **Tae Min Kim:** writing – review and editing, investigation, resources, data curation. **Chang Gon Kim:** writing – review and editing, investigation, resources, data curation. **Hee Kyung Ahn:** writing – review and editing, investigation, resources, data curation. **Youngjoo Lee:** writing – review and editing, investigation, resources, data curation. **Yu Jung Kim:** writing – review and editing, investigation, resources, data curation. **Miso Kim:** writing – review and editing, investigation, resources, data curation. **Jeonghwan Youk:** writing – review and editing, investigation, resources, data curation. **Jong‐Mu Sun:** writing – review and editing, investigation, resources, data curation. **Se‐Hoon Lee:** writing – review and editing, investigation, resources, data curation. **Jin Seok Ahn:** writing – review and editing, investigation, resources, data curation. **Dong‐Wan Kim:** writing – review and editing, investigation, resources, data curation. **Myung‐Ju Ahn:** writing – review and editing, investigation, resources, data curation. **Yoon Ji Choi:** writing – review and editing, investigation, resources, data curation. **Sun Min Lim:** writing – review and editing, investigation, resources, data curation. **Jin Hyoung Kang:** writing – review and editing, investigation, resources, data curation. **Bhumsuk Keam:** writing – review and editing, writing – original draft, supervision, investigation, resources, data curation, conceptualization. **Hye Ryun Kim:** writing – review and editing, writing – original draft, supervision, investigation, resources, data curation, conceptualization.

## Funding

This study was supported by the National R&D Program for Cancer Control through the National Cancer Center (NCC) funded by the Ministry of Health & Welfare, Republic of Korea (RS‐2022‐CC125163).

## Conflicts of Interest

M.H. Hong received honoraria from AstraZeneca, Amgen, BMS, MSD, Ono Pharmaceutical, Takeda, and Roche; served in a consulting or advisory role for AstraZeneca, BMS, MSD, Pfizer, Takeda, Roche, and Yuhan; acted as an investigator or co‐investigator of trials for Abbvie, AstraZeneca, BMS, IMPACT Therapeutics, Ignyta, Loxo Oncology, Merck Sereno, MSD, Novartis, ORIC, Roche, Pfizer, and Yuhan; and received research support from AstraZeneca, MSD, Novartis, and Yuhan. C.G. Kim reported consulting or advisory roles from Astellas, Amgen, BeOne, Johnson & Johnson/Janssen, MSD Oncology, Novartis, Ono Pharmaceutical, and Pfizer, speakers' bureau from Astellas, AstraZeneca, BeOne, Dong‐A ST, Hanmi, Merck, MSD Oncology, and Novartis, and research funding from Astellas, Exelixis, and Ipsen. D‐W. Kim reports received research funding to his institution from Alpha Biopharma, Amgen, AstraZeneca, BMS, Boehringer‐Ingelheim, Bridge BioTherapeutics, Chong Keun Dang, Daiichi‐Sankyo, GSK, Hanmi, IMBDx, InnoN, IQVIA, Janssen, Merck, Merus, Mirati Therapeutics, MSD, Novartis, Ono Pharmaceutical, Pfizer, Roche/Genentech, Takeda, TP Therapeutics, Xcovery, Yuhan, and Zymeworks; and received medical writing assistance from Amgen, AstraZeneca, BMS, Boehringer‐Ingelheim, Bridge BioTherapeutics, Chong Keun Dang, Daiichi‐Sankyo, GSK, Hanmi, IMBDx, Janssen, Merck, Merus, Mirati Therapeutics, MSD, Novartis, Pfizer, Riegel Pharmaceuticals, Roche/Genentech, Takeda, Yuhan, and Zymeworks. S.M. Lim reports personal grants from Yuhan, Merck Sharp & Dohme and Johnson and Johnson; institutional grants from Yuhan, Boehringer Ingelheim, BridgeBio Therapeutics, Roche, GSK, Jiangsu Hengrui, AstraZeneca, Lily, Takeda, Daiichi Sankyo, J Ints Bio, Guardant, BeOne, BioNTech, Johnson and Johnson, Therapex, Oscotec, Merck, Bristol Myers Squibb, Merck Sharp & Dohme and Amgen. B. Keam received research funding from MSD, AstraZeneca, Bayer, and Ono Pharmaceutical Co. Ltd., and has served as an advisor for Handok, Yuhan, Trialinformatics, TiumBio, and BeOne outside of the current work. H.R. Kim received clinical trial funding to her institution from AbbVie, ABL Bio, AstraZeneca, Bayer, BeiGene, Boehringer Ingelheim, Boryung, Bristol Myers Squibb, F. Hoffmann‐La Roche Ltd./Genentech Inc., Hanmi, Genmab, GSK. Merck & Co. Inc., Pfizer, Regeneron, Samsung Bioepis, Seagen, Takeda, and Yuhan; participated in speaker bureaus for AstraZeneca, MSD, Takeda, Yuhan; and served on advisory boards for AstraZeneca, Daichi, Janssen, and Takeda.

## Supporting information


**Figure S1:** Kaplan–Meier curves of survival outcomes based on study: LU20‐15 versus LU21‐01. (A) Overall survival; (B) progression‐free survival; (C) intracranial progression‐free survival.
**Figure S2:** Kaplan–Meier curves of survival outcomes based on EGFR mutation: exon 19 deletion versus L858R mutation. (A) Overall survival; (B) progression‐free survival; (C) intracranial progression‐free survival.
**Figure S3:** Kaplan–Meier curves of survival outcomes based on type of central nervous system involvement. (A) Overall survival; (B) progression‐free survival; (C) intracranial progression‐free survival.
**Figure S4:** Kaplan–Meier curves of survival outcomes based on T790M status. (A) Overall survival; (B) progression‐free survival; (C) intracranial progression‐free survival.
**Table S1:** Baseline characteristics according to study.
**Table S2:** Response assessments according to study.
**Table S3:** Baseline characteristics according to T790M status.

## Data Availability

The data that support the findings of this study are available from the corresponding author upon reasonable request.
