# Supplementary material for: Central Nervous System Activity of Lazertinib in EGFR ‐Mutated NSCLC With Prior EGFR TKI Exposure: A Pooled Analysis of KCSG‐LU20‐15 and LU21‐01
Source: Thorac Cancer. 2026 Aug 31;17(17):e70387. doi: 10.1111/1759-7714.70387 (PMC13527518; doi:10.1111/1759-7714.70387)
Supplement: Supplementary file 1 [file TCA-17-e70387-s001.docx]

**SUPPLEMENTARY MATERIAL**

**Central Nervous System Activity of Lazertinib in *EGFR*-mutated NSCLC with Prior EGFR TKI Exposure: A Pooled Analysis of KCSG-LU20-15 and LU21-01**

Dong Hyun Kim^*^, Min Hee Hong^*^, Hyun Ae Jung, Tae Min Kim, Chang Gon Kim, Hee Kyung Ahn, Youngjoo Lee, Yu Jung Kim, Miso Kim, Jeonghwan Youk, Jong-Mu Sun, Se-Hoon Lee, Jin Seok Ahn, Dong-Wan Kim, Myung-Ju Ahn, Yoon Ji Choi, Sun Min Lim, Jin Hyoung Kang, Bhumsuk Keam^*^, Hye Ryun Kim^*^

*DHK and MHH made equal contribution

*BK and HRK equal contribution

**Correspondence to:**

Bhumsuk Keam, MD, PhD

Department of Internal Medicine, Seoul National University Hospital, 101, Daehak-ro, Jongro-gu, Seoul 03080, Republic of Korea

Tel +82-2-2072-7215

E-mail [bhumsuk@snu.ac.kr](mailto:bhumsuk@snu.ac.kr)

Hye Ryun Kim, MD, PhD

Medical Oncology, Department of Internal Medicine, Yonsei Cancer Center, Severance Hospital, Yonsei University College of Medicine, 50 Yonsei-ro, Seodaemun-gu, Seoul, Republic of Korea

E-mail [nobelg@yuhs.ac](mailto:nobelg@yuhs.ac)

**Supplementary Methods**

**Study treatment**

For the KCSG-LU20-15 study (NCT05326425), patients received lazertinib 240 mg once daily. Each treatment cycle lasted for 42 days. Dose reduction to 160 mg was permitted in patients who experienced adverse events while receiving the 240 mg dose.

For the KCSG-LU21-01 study (KCT0005919), patients received lazertinib 240 mg once daily, in combination with pemetrexed (500 mg/m^2^) every 3 weeks. If patients experienced no serious adverse events and remained clinically stable, the lazertinib dose could be escalated to 320 mg at the investigator’s discretion. Conversely, in cases of grade ≥3 toxicities deemed related to lazertinib, or other unacceptable adverse events, treatment was temporarily interrupted and the dose was reduced to 160 mg; dose reductions below 160 mg were not allowed.

**Response evaluation**

For the KCSG-LU20-15 study (NCT05326425), tumor response was assessed according to the RECIST v 1.1 after every treatment cycle during the first 4 assessments and every two cycles thereafter. For the KCSG-LU21-01 study (KCT0005919), disease progression was defined by any of the following: (1) radiologic progression of intracranial lesions or significant radiologic progression of LM on brain MRI according to RECIST v 1.1; (2) extracranial disease progression according to RECIST v 1.1; or (3) reappearance of malignant cells on CSF cytology after a previous negative cytologic result. Radiologic assessments, including brain MRI and chest and abdominal CT, were performed every 9 weeks. CSF cytology was assessed at baseline and at cycles 2 and 3.

**Supplementary Figures**

**Figure S1.** Kaplan-Meier curves of survival outcomes based on study: LU20-15 vs. LU21-01. (A) Overall survival; (B) Progression-free survival; (C) Intracranial progression-free survival.

**Figure S2.** Kaplan-Meier curves of survival outcomes based on EGFR mutation: exon 19 deletion vs. L858R mutation. (A) Overall survival; (B) Progression-free survival; (C) Intracranial progression-free survival.

**Figure S3.** Kaplan-Meier curves of survival outcomes based on type of central nervous system involvement. (A) Overall survival; (B) Progression-free survival; (C) Intracranial progression-free survival.

**Figure S4.** Kaplan-Meier curves of survival outcomes based on T790M status. (A) Overall survival; (B) Progression-free survival; (C) Intracranial progression-free survival.

**Supplementary Tables**

**Table S1**. Baseline characteristics according to study.

| **Variables** | **LU20-15**  **N = 38** | **LU21-01**  **N = 34** | ***P*-value** |
| --- | --- | --- | --- |
| **Age, years, median (range)**  Age ≥ 65, *n* (%) | 64 (29–85)  19 (50.0) | 62 (38–80)  12 (35.3) | 0.756  0.308 |
| **Sex, *n* (%)**  Male  Female | 15 (39.5)  23 (60.5) | 14 (41.2)  20 (58.8) | 0.999 |
| **ECOG performance status, *n* (%)**  0  1  2 | 6 (15.8)  30 (78.9)  2 (5.3) | 2 (5.9)  21 (61.8)  11 (32.4) | 0.008 |
| **Smoking history, *n* (%)**  Never smoker  Ex-smoker  Current smoker | 25 (65.8)  12 (31.6)  1 (2.6) | 24 (70.6)  10 (29.4)  0 | 0.612 |
| **Histology, *n* (%)**  Adenocarcinoma  Others | 35 (92.1)  3 (7.9) | 32 (94.1)  2 (5.9) | 0.999 |
| **EGFR variants prior to previous EGFR TKIs, *n* (%)**  Exon 19 deletion  L858R  Others | 18 (47.4)  20 (52.6)  0 | 11 (32.4)  20 (58.8)  3 (8.8) | 0.106 |
| **Prior EGFR TKI usage, *n* (%)**  Gefitinib  Afatinib  Erlotinib  Dacomitinib | 17 (44.7)  17 (44.7)  5 (13.2)  0 | 17 (50.0)  14 (41.2)  7 (20.6)  1 (2.9) | 0.834  0.947  0.598  0.955 |
| **Type of CNS lesion, *n* (%)**  Brain metastasis only  Leptomeningeal metastasis ± brain metastasis | 27 (71.1)  11 (28.9) | 0  34 (100.0) | < 0.001 |
| **Other metastatic sites, *n* (%)**  Bone  Lymph node  Pleura  Liver  Adrenal | 19 (50.0)  15 (39.5)  7 (18.4)  3 (7.9)  2 (5.3) | 14 (41.2)  16 (47.1)  4 (11.8)  2 (5.9)  1 (2.9) | 0.608  0.681  0.649  0.999  0.999 |

CNS, central nervous system; ECOG, Eastern Cooperative Oncology Group; EGFR, epidermal growth factor receptor; TKI, tyrosine kinase inhibitor.

**Table S2**. Response assessments according to study.

| **Variables** | **LU20-15**  **(Lazertinib)**  **N = 38** | **LU21-01**  **(Lazertinib + Pemetrexed)**  **N = 34** |
| --- | --- | --- |
| **Overall response, *n* (%)**  Complete response  Partial response  Stable disease  Progressive disease | N = 38  0  8 (21.1)  29 (76.3)  1 (2.6) | N = 28  0  7 (25.0)  20 (71.4)  1 (3.6) |
| **Overall ORR, % (95% CI)** | 21.1 (9.6–37.3) | 25.0 (10.7–44.9) |
| **Overall DCR, % (95% CI)** | 97.4 (86.2–99.9) | 96.4 (81.7–99.9) |
|  |  |  |
| **Intracranial response, *n* (%)**  Complete response  Partial response  Stable disease  Progressive disease | N = 38  2 (5.3)  19 (50.0)  16 (42.1)  1 (2.6) | N = 8  0  2 (25.0)  6 (75.0)  0 |
| **Intracranial ORR, % (95% CI)** | 55.3 (38.3–71.4) | 25.0 (3.2–65.1) |
| **Intracranial DCR, % (95% CI)** | 97.4 (86.2–99.9) | 100 (63.1–100) |
|  |  |  |
| **Extracranial response, *n* (%)**  Complete response  Partial response  Stable disease  Progressive disease | N = 36  0  7 (19.4)  28 (77.8)  1 (2.8) | N = 20  0  5 (25.0)  14 (70.0)  1 (5.0) |
| **Extracranial ORR, % (95% CI)** | 19.4 (8.2–36.0) | 25.0 (8.7–49.1) |
| **Extracranial DCR, % (95% CI)** | 97.2 (85.5–99.9) | 95.0 (75.1–99.9) |

ORR, objective response rate; DCR, disease control rate; CI, confidence interval.

**Table S3**. Baseline characteristics according to T790M status.

| **Variables** | **T790M-positve**  **N = 7** | **T790M-negative**  **N = 44** | ***P*-value** |
| --- | --- | --- | --- |
| **Overall response, *n* (%)**  Complete response  Partial response  Stable disease  Progressive disease | N = 7  0  2 (28.6)  5 (71.4)  0 | N = 40  0  9 (22.5)  29 (72.5)  2 (5.0) | 0.801 |
| **Overall ORR, % (95% CI)** | 28.6 (3.7–71.0) | 22.5 (10.8–38.5) | 0.999 |
| **Overall DCR, % (95% CI)** | 100 (59.0–100) | 95.0 (83.1–99.4) | 0.999 |
|  |  |  |  |
| **Intracranial response, *n* (%)**  Complete response  Partial response  Stable disease  Progressive disease | N = 6  0  5 (83.3)  1 (16.7)  0 | N = 25  1 (4.0)  10 (40.0)  13 (52.0)  1 (4.0) | 0.296 |
| **Intracranial ORR, % (95% CI)** | 83.3 (35.9–99.6) | 44.0 (24.4–65.1) | 0.202 |
| **Intracranial DCR, % (95% CI)** | 100 (54.1–100) | 96.0 (79.6–99.9) | 0.999 |
|  |  |  |  |
| **Extracranial response, *n* (%)**  Complete response  Partial response  Stable disease  Progressive disease | N = 6  0  1 (16.7)  5 (83.3)  0 | N = 35  0  8 (22.9)  25 (71.4)  2 (5.7) | 0.767 |
| **Extracranial ORR, % (95% CI)** | 16.7 (0.4–64.1) | 22.9 (10.4–40.1) | 0.999 |
| **Extracranial DCR, % (95% CI)** | 100 (54.1–100) | 94.3 (80.8–99.3) | 0.999 |

ORR, objective response rate; DCR, disease control rate; CI, confidence interval.
